# DecisionDx-GBM Gene Expression Assay for Prognostic Testing in Glioblastoma Multiform

**DOI:** 10.1371/currents.RRN1186

**Published:** 2010-11-26

**Authors:** Diane Allingham-Hawkins, Andrew Lea, Susan Levine

**Affiliations:** Hayes, Inc.

## Abstract

It is estimated that approximately 22,000 Americans will be diagnosed with tumor of the brain or nervous system in 2010. Among primary brain tumors, approximately 60% are gliomas, the most common and most malignant of which is glioblastoma multiforme (GBM).The DecisionDx-GBM test is a multigene expression assay that is designed to predict which patients are likely to experience long-term (> 2 years) progression-free survival.

## **Clinical Scenario**

In 2010, approximately 22,000 Americans will be diagnosed with a tumor of the brain or nervous system [1]. Glioblastoma multiforme (GBM) are the most common primary brain tumors. GBMs are aggressive tumors and despite improvements in treatment regimens, which include surgical resection, radiation, and chemotherapy, prognosis is poor with a median survival of 14.6 months[2] Research has indicated that the genomes of GBMs have multiple changes including deletion of tumor suppressor genes and amplification or over-expression of tyrosine kinase receptors leading to both survival advantage and apoptosis resistance in tumor cells. Consequently, it has been suggested that any successful targeted therapy must take into account multiple genomic changes simultaneously[2].  The DecisionDx-GBM test is a multigene expression assay that is designed to predict which patients are likely to experience long-term (> 2 years) progression-free survival[3].

## **Test Description**

The DecisionDx-GBM test uses formalin-fixed, paraffin-embedded (FFPE) tumor tissue. RNA is extracted from this specimen and used to measure the expression of 9 genes reported to be associated with survival including 7 genes correlated with decreased survival (aquaporin 1 [*AQP1*], chitinase 3-like 1 [*CHI3L1*], epithelial membrane protein 3 [*EMP-3*], glycoprotein NMB [GPNMB], insulin-like growth factor-binding protein 2 [*IGFBP2*], galectin 3 [*LGALS3*] and podoplanin [*PDPN*]) and 2 genes associated with improved survival (oligodendrocyte lineage transcription factor 2 [*OLIG2*] and reticulon 1 [*RTN1*]) as well as 3 control genes (eukaryotic translation elongation factor 1, alpha-1 [*EEF1A1*]; beta-glucoronidase [*GUSB*]; and ribosomal protein S27 [*RPS27*])[4]. A proprietary algorithm is used to convert expression levels to a DecisionDx-GBM score, which is then compared to an underlying clinical database. Results are reported as both a DecisionDx-GBM score and a quintile rank compared to other patients in the database. The likelihood of 2-year survival is provided, along with the expected median survival and median progression-free survival[3] [4] [5].

## **Public Health Importance**

Although standard histo-pathological methods can accurately diagnosis GBM, no information on patient prognosis is provided. Having prognostic information may lead to a change in patient management such that more aggressive treatments are used earlier in patients with a poorer predicted prognosis. However, it is important to note that there have been no demonstrations that the use of a gene expression assay such as DecisionDx-GBM to guide management of patients with GBM results in improved patient outcomes. 

## **Published Reviews, Recommendations and Guidelines** 

**Systematic evidence reviews**

None identified.

**Recommendations by independent group**

None identified. 

**Guidelines by professional groups**

None identified.

## **Search Strategy** 

- Searches of MEDLINE and EMBASE were conducted on September 16, 2010, using the search terms (*glioblastoma* OR *GBM*) AND (*AQP1* OR *aquaporin 1*) AND (*CHI3L1* OR *chitinase 3-like 1* OR *YKL-40*) AND (*EMP-3* OR *epithelial membrane protein 3*) AND (*GPNMB* OR *glycoprotein NMB*) AND (*IGFBP2* OR *insulin-like growth factor binding protein 2*) AND (*LGALS3* OR *galectin 3*) AND (*OLIG2* OR *oligodendrocyte lineage transcription factor 2*) AND (*PDPN* OR *podoplanin*) AND (*RTN1* OR *reticulon 1*); (*glioblastoma* OR *GBM*) AND (*AQP1* OR *aquaporin 1* OR *CHI3L1* OR *chitinase 3-like 1* OR *YKL-40* OR *EMP-3* OR *epithelial membrane protein 3* OR *GPNMB* OR *glycoprotein NMB* OR *IGFBP2* OR *insulin-like growth factor binding protein 2* OR *LGALS3* OR *galectin 3* OR *OLIG2* OR *oligodendrocyte lineage transcription factor 2* OR *PDPN* OR *podoplanin* OR *RTN1* OR *reticulon 1*); and *Colman H* (au). Limits used were human, English language, and published since January 1, 1996.
- The above search strategies yielded 0, 126, and 53 citations, respectively. Only a single study directly related to the DecisionDx-GBM test was identified.

## **Evidence Overview** 

***Analytic Validity*:**Test accuracy and reliability in measuring expression of 9 survival and 3 control genes (analytic sensitivity and specificity).

No information regarding the analytical validity of the DecisionDx-GBM assay was identified in either the primary publication or on the Castle Biosciences website [3] [5]

***Clinical Validity*:**Test accuracy and reliability in accurately predicting patient prognosis (predictive value).

- Gene expression data derived from expression microarray studies on frozen tumors specimens from a total of 110 patients from 4 institutions (Massachusetts General Hospital, M.D. Anderson Cancer Center, University of California Los Angeles and University of California San Francisco) were used to identify 38 genes whose expression was significantly associated with survival[5].
- A reverse-transcription polymerase chain reaction (RT-PCR) assay for the 38 genes was developed and validated on a separate set of 68 FFPE GBM tumor specimens. From this validation, the 9 genes with the highest survival associations were selected[5].
- The 9-gene assay was validated on a final set of 101 FFPE GBM tumor specimens. Expression of these 9 genes was found through multivariate analysis to be an independent predictor of progression-free survival (Cox proportional hazard ratio [HR] = 2.7; P=0.0003) and overall survival (Cox HR = 2.7; P=0.0003) compared to age, performance score, and methylation status of the methylguanine methyltransferase gene (MGMT). MGMT methylation has previously been reported to be an independent predictor of response of GBM to radiation and chemotherapy[6]. Interestingly, MGMT methylation was not found to be an independent predictor of survival in the DecisionDx-GBM validation studies[5].

***Clinical Utility*: **Net benefit of test in improving health outcomes.

- No published studies demonstrating improved health outcomes were identified.
- Two clinical trials have the DecisionDx-GBM test as part of the study as either a risk stratification method or as a secondary endpoint. These studies are:

- Temozolomide and Radiation Therapy With or Without Bevacizumab in Treating Patients With Newly Diagnosed Glioblastoma (NCT00884741)
- Radiation Therapy and Temozolomide in Treating Patients With Newly Diagnosed Glioblastoma or Gliosarcoma (NCT00304031)

## **Limitations**

- Specimen numbers - to date the current version of the DecisionDx-GBM assay has only been validated on 101 specimens. Validation with a larger data set is needed to ensure that preliminary findings are consistent.
- Lack of transparency - the proprietary algorithm used to generate the risk score has not been independently validated.

## **Conclusion**

There is currently only a single peer-reviewed publication on the derivation and validation of this assay. No information is provided on the analytical validity of the assay. No studies on the clinical utility of this test in the care of patients with GBM have been published. Therefore, there is currently insufficient evidence to recommend adoption of this test for routine use in the care of patients with GBM.

## **Links**

None identified.

Last updated:October 8, 2010

## **Acknowledgments**

The authors would like to acknowledge the contributions of the members of the Hayes Genetic Test Evaluation team, particularly Lisa Spock, Linnie Wieselquist and Charlotte Kuo-Benitez. 

## **Funding information**

Funding for the Health Technology Assessment that informed this work was provided by Hayes, Incorporated. Funding to create this Knol was provided by the Centers for Disease Control and Prevention under Contract No. 200-2009-F-32675.This funding was provided through the Genetic Alliance.

## **Competing interests**

The authors are employees at Hayes, Inc., an independent health technology research and consulting company. None of the employees at this company has any financial or personal interest in any of the technologies reviewed by Hayes, Inc.. No input on report content or conclusions is permitted by manufacturers. Although the CDC funded the work to produce this article, the content is based entirely on Hayes, Inc.'s own analysis and there was no input from the CDC.
